# A compact, modular and low‑cost hydroponic greenhouse

**DOI:** 10.1016/j.ohx.2026.e00777

**Published:** 2026-04-26

**Authors:** Teresa Iucci, Dren Maliqi, Sara Sousa Rosa, Marco P.C. Marques

**Affiliations:** a Department of Biochemical Engineering, University College London, Gordon Street, London WC1E 6BT, United Kingdom; b High Precision Design and Fabrication Facility, University College London, Torrington Place, London WC1E 7JE, United Kingdom; c Department of Chemistry and Pharmaceutical Technologies, University La Sapienza, Piazzale Aldo Moro 5, 00185 Rome, Italy

**Keywords:** Greenhouse, Hydroponic system, Plant molecular farming, *Nicotiana benthamiana*, Sustainable, Controlled-environment agriculture (CEA)

## Abstract

Plant Molecular Farming (PMF) is emerging as a cost-effective alternative for producing recombinant biopharmaceuticals, yet laboratory-scale cultivation platforms remain limited. We present a compact, modular hydroponic greenhouse designed to support supervised growth of *Nicotiana benthamiana,* or other plant species, in standard laboratory environments. The system integrates a recirculating hydroponic module, rockwool-based plant support, adjustable LED lighting, and continuous monitoring of key environmental parameters, ensuring reproducible and physiologically suitable growth conditions. Validation across four cultivation batches demonstrated stable temperature (23–25 °C), relative humidity (∼60%), and uniform light distribution, resulting in consistent plant growth and morphology (maximum height: 23 cm, total leaf area: 420 cm^2^, and leaf mass: 489 g). With a low build cost (£781) and scalable, open-source design, this platform provides an accessible solution for early-stage PMF research and upstream process development.

## Highlights

• Compact hydroponic greenhouse providing supervised growth of *N. benthamiana.*
• Rockwool-based hydroponic system for stable and reproducible cultivation.
• Validated light, temperature and humidity conditions for uniform plant growth.
• Low-cost system (≈£781) suitable for early-stage biopharmaceutical studies.

## Graphical abstract

**Figure:**
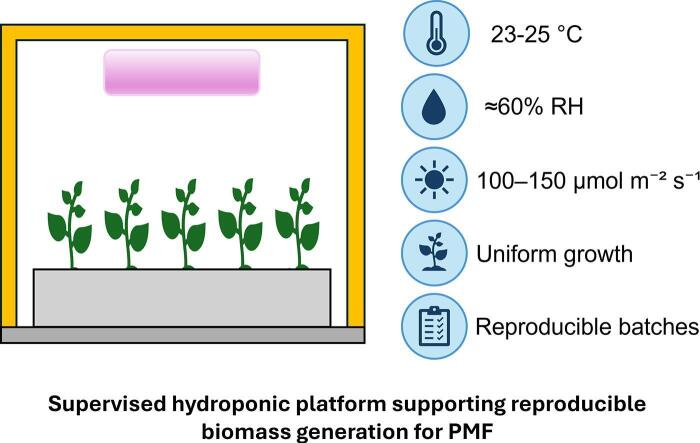

**Specifications table**

**Table:** 

Hardware name	*Small Scale Hydroponic Greenhouse*
Subject area	• Engineering and materials science • Chemistry and biochemistry • Biological sciences
Hardware type	• Field measurements and sensors • Biological sample handling and preparation • Other [hydroponic system with integrated with LED lamp and recirculation pump]
Closest commercial analog	No direct commercial analog is available.The closest system is the Sananbio Radix platform (sananbiofarm.com), which is designed for large-scale industrial cultivation. Other existing hydroponic units do not provide the controlled-environment features required for laboratory PMF studies (e.g. https://amzn.eu/d/auCqGa6, https://amzn.eu/d/dZjGpvD)
Open source license	CC BY-NC
Cost of hardware	∼ £781 (€903, $1040)
Source file repository	http://doi.org/10.17632/kn3ysjkrsp.2

## 1 Hardware in context

Demand for recombinant biopharmaceuticals has increased rapidly, often exceeding the production capacity of traditional microbial and mammalian expression platforms [1]. Therefore, it is essential to adopt alternative production systems that can deliver scalability, lower costs, and rapid solutions. Over the past two decades, this need has led to the rise of Plant Molecular Farming (PMF) [2] and the development of several cultivation platforms in which plants, especially *Nicotiana benthamiana*, are used as flexible and efficient bioreactors [3], [4]. *N. benthamiana* has become the preferred host due to its high biomass yield, susceptibility to *Agrobacterium*-mediated transient expression, ease of genetic engineering, and its compatibility with pharmaceutical production workflows [5], [6]. PMF offers several intrinsic advantages, including significantly reduced production costs [7]. Traditional fermenters and bioreactors used in conventional biotech processes can be replaced by controlled plant-growth environments, which exclude human-pathogenic contaminants and therefore lower initial infrastructure requirements [8].

Within PMF, cultivation conditions play a fundamental role in ensuring uniform plant growth, morphology, and recombinant protein expression [9]. Controlled-environment greenhouses are increasingly adopted, as they limit cross-contamination with other crops, reduce exposure to external environmental fluctuations, and do not require sterile conditions as plants are not susceptible to pathogens harmful to humans [10]. Plant cultivation rely on traditional soil-based methods or soilless hydroponic approaches. Hydroponics offers greater control over nutrient delivery by supplying minerals directly to the roots in ionic form, enabling more precise and adaptable cultivation across the plant’s growth cycle [11]. This results in accelerated biomass development and more homogeneous growth. Hydroponic cultivation also eliminates soil-borne pathogens, reduces pesticide use, supports automation and decreases water consumption [12]. Key parameters, including pH (5.8–6.5), electrical conductivity (1.5–2.5 dS m^−1^), nutrient composition, and adequate aeration, directly influence the reproducibility and stability of upstream PMF workflows [11], [13]. Despite the initial investment required to establish the greenhouse, hydroponics provides a more sustainable and resource-efficient strategy compared to conventional soil-based cultivation.

Current commercially available hydroponic platforms do not fully meet the specific needs of laboratory-scale plant cultivation. Large-scale industrial systems, such as the Sananbio Radix modular platform, provide advanced environmental control but require substantial physical space and capital investment [14]. In contrast, small-scale hydroponic units lack controlled lighting, environmental monitoring, and GMP-aligned features, making them unsuitable for upstream bioprocess development. This highlights a necessity for compact, modular, and reproducible systems that can be integrated into GMP-compatible laboratory settings [2], [10], [15].

The greenhouse presented in this work was designed to address this gap by providing a laboratory-scale system that is cost-effective, suitable for preliminary studies, and equipped with features required for pharmaceutical-grade plant cultivation. It supports the hydroponic growth of up to fifteen *N. benthamiana* plants under supervised and consistently maintained conditions. The unit integrates a recirculating nutrient solution enriched with oxygen, a rockwool-based support structure, LED lighting with a photoperiod optimised for vegetative growth, and continuous monitoring of key environmental variables. To ensure air renewal and reduce exposure to external contaminants, the system is intended to operate inside a laboratory fume hood. Compact and low-cost, this platform offers an accessible solution for early-stage PMF research while aligning with core principles of controlled‑environment cultivation, achieved here through monitoring and manual adjustment rather than automated regulation.

## 2 Hardware description

**Fig. 1:**
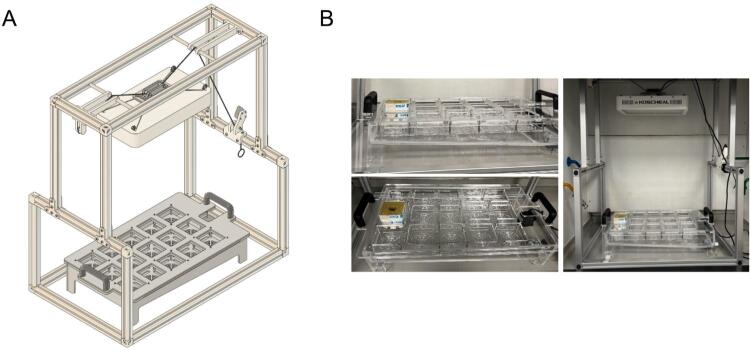
Model of the laboratory-scale cultivation system used for plant growth. (A) Technical drawing showing the nutrition solution tray, a plant-support grid, and an external frame housing the LED illumination system. (B) Experimental setup showing the cultivation trays designed to accommodate up to fifteen *N. benthamian*a plants and the system integration within a controlled fume hood environment.

The compact, laboratory-scale hydroponic greenhouse is designed to enable controlled cultivation of N. benthamiana for PMF applications. It comprises a modular growing unit that integrates a nutrient-solution tray, a plant-support grid, and an external frame housing the LED illumination system (Fig. 1).

The frame is constructed from anodized aluminium, chosen for its affordability, corrosion resistance, and lightweight properties, which enhance portability and ease of handling while ensuring structural stability. Its modular design and compatibility with standard fasteners make it particularly suitable for laboratory-scale systems that require frequent handling and/or relocation. The frame is assembled using screw connections, providing a rigid structure and integrated supports for the LED illumination system. The total weights of the frame and the LED illumination system are 10.2 kg and 2.5 kg, respectively. Overall dimensions are 90 cm (height) × 82 cm (width) × 50 cm (depth), allowing the unit to fit within a standard laboratory fume hood (e.g. Lab serv 1200).

The cultivation tray serves as the reservoir for the hydroponic nutrient solution and incorporates a sloped bottom to enable complete drainage, facilitate cleaning, and allow rapid solution exchange. The trays are fabricated from polymethyl methacrylate (PMMA, acrylic), selected for its low cost, mechanical resistance, and optical transparency, which allows visual inspection of root development, monitoring of the nutrient solution and assessment of its consumption or evaporation. The tray measures 59 cm × 32.5 cm × 12 cm (width × depth × height). The bottom surface of the tray features a vertical offset of 21 cm, corresponding to a slope ratio of 0.356 and an inclination angle of ∼19.6°, ensuring efficient drainage through a ball valve positioned at the lowest point. The empty tray weighs 2.7 kg, and when filled with nutrient solution it has a total weight of 5.7 kg. All structural components were assembled using adhesive bonding to guarantee leak-tight integrity and mechanical stability.

The plant support grid is designed to hold rockwool cubes measuring 7.8 cm × 7.8 cm × 6.6 cm (width × depth × height), a substrate that has high oxygen retention capacity and suitability for root development [16]. On either side of the support grid, two handles are integrated (bringing total height to 18 cm) to enable easy removal during cleaning operations or plant inspection. The grid layout was designed to accommodate space for fifteen plants with a uniform spacing of 3 cm between them to promote homogeneous leaf expansion and minimise shading. The plant support grid weighs 2 kg when no plants are present. A dedicated side area in the tank (6 cm × 5 cm), positioned outside the main light beam, accommodates a submersible pump (to ensure continuous recirculation and homogeneous oxygenation of the root zone [17]), the pH and conductivity electrodes, and an opening for nutrient-solution additions, if necessary.

The aluminium frame supports the LED lamp, whose height can be adjusted to maintain a constant distance from the plant canopy throughout the growth cycle (between 20 and 25 cm). The selected LED unit provides wavelengths (396 to 730 nm), a spectrumoptimized for *N. benthamiana* vegetative growth, but also suitable for supporting the growth of many other plant species. It additionally advantages over fluorescent lighting, including lower heat generation, longer operational lifespan, and reduced energy consumption [18]. Overall, the dimensions of the growing module and the position of the LED lamp were designed to ensure uniform illumination, enabling all plants to receive comparable light intensity.

The system is designed for operation within a standard fume hood, where closing the sash and maintaining a front lip barrier helps to minimise the intake of external contaminants, such as dust particulates, microbial contaminants, and environmental aerosols. Under these conditions, the use of a recirculating fume hood is not required. Its modular architecture enables scalability and customization: grid dimensions and spatial configuration can be adapted to accommodate diverse plant species, while process-specific requirements can be addressed through integration of additional sensing elements and environmental monitoring modules.

This hardware platform supports:

– Laboratory-scale cultivation experiments aimed at the production of plant-derived biopharmaceuticals or others.
– Supervised hydroponic growth optimization of *N. benthamiana* through consistent environmental monitoring and manual adjustments, with system parameters suitable for supporting the cultivation of a wide range of other plant species.
– Upstream process development for agroinfiltration and transient-expression workflows, enabling reproducible and scalable studies.

### 2.1 Control and acquisition system

The hydroponic growth system was designed to support N. benthamiana cultivation by providing a supervised an consistently monitored environment. Temperature, pH, and electrical conductivity (EC) of the nutrient solution were periodically measured and adjusted manually as needed to maintain suitable growth conditions. Humidity was supported using a portable humidifier (Bear 5 L humidifier), which helped keep relative humidity within the desired range to minimise excessive transpiration and reduce the risk of fungal growth.

Environmental parameters were monitored using an ElitechLog V8.0 (external temperature and humidity), and Mettler Toledo SevenDirect with pH and EC probes. These sensors provided data which enabled precise adjustment of nutrient composition. Temperature of the nutrient solution was recorded using a ElitechLog V8.0 placed in the nutrient reservoir. Light intensity was monitored using Elitech digital Luxmeter on a regular basis.

Sensor placement was optimised to minimise disturbance and ensure accurate readings: pH and EC probes were immersed in the nutrient solution, thermocouples were positioned near the root zone, and humidity sensors were located at canopy level. Data were sampled at 5-minute intervals and stored in a structured database for subsequent analysis, although processing using standard spreadsheet software (e.g. Microsoft Excel) is also sufficient.

This configuration provides a cost-effective and modular solution for supervised hydroponic growth, enabling consistent environmental monitoring, manual adjustment, and improved reproducibility in plant development studies. Future improvements could include integrating machine‑learning assisted decision tools for predictive guidance and developing automated nutrient‑delivery systems to reduce manual intervention.”

## 3 Design files

**Fig. 2:**
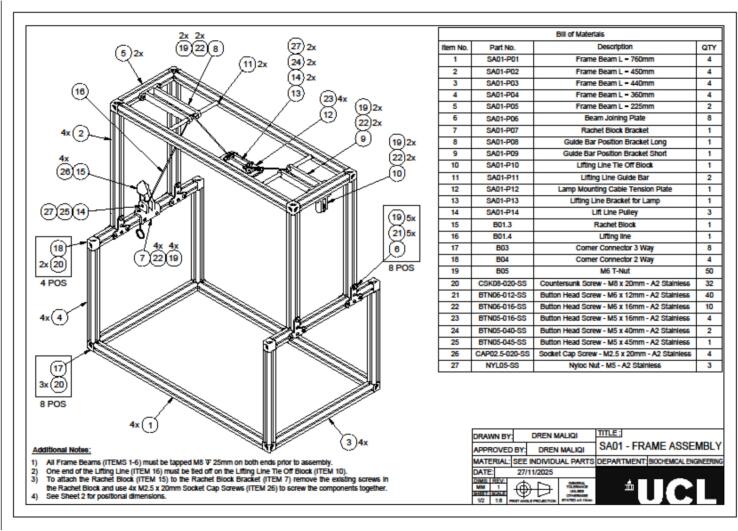
Isometric view of the structural frame for the lighting system, showing aluminium beams, joining plates, brackets, pulleys, and connectors. The diagram includes part numbers and quantities as listed in the Bill of Materials (Table 3), along with assembly notes for tapping and fastening components.

**Fig. 3:**
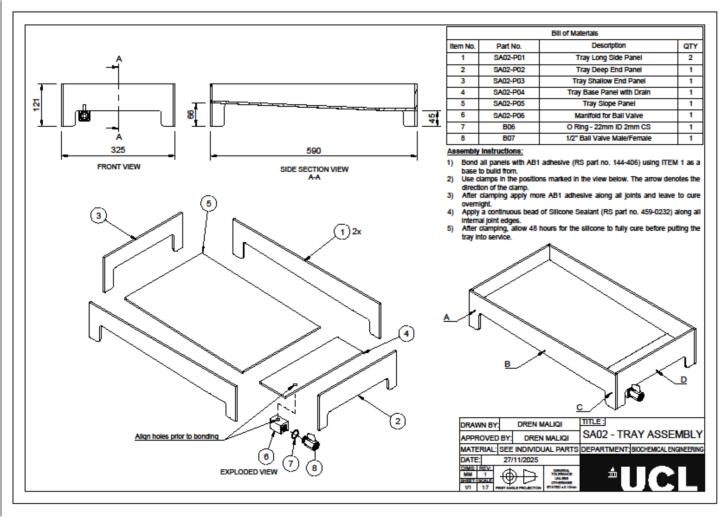
Technical drawing of the tray assembly showing front view, side section, and exploded view with labelled components and bill of materials.

The hydroponic greenhouse was designed using Autodesk Fusion 360. All design files are provided in.STEP format, accompanied by technical drawings in.PDF format to support fabrication. These include the complete greenhouse structure (file Small scale hydroponic greenhouse.step and Fig. 5), the frame, the tray, and corresponding inserts. Detailed diagrams of all components are shown in Fig. 2, Fig. 3. The files offer a comprehensive structural and functional layout for fabrication, assembly, and replication of the system, enabling users to inspect individual parts, modify dimensions, and reproduce the device accurately. All files are available in an online repository (Mendeley Data) and summarised in Table 1.

**Table 1:** Summary of design files available in the repository.

Design file name	File type	Location of the file
Small scale hydroponic greenhouse	.step file	Mendeley data
SA01|Frame Assembly v15:1	PDF	Fig. 2, Medeley data
SA02|Tray Assembly v15:1	PDF	Fig. 3, Medeley data
SA03|Tray Insert Assembly	PDF	Fig. 4, Medeley data

## 4 Bill of materials summary

The construction of the small-scale hydroponic greenhouse requires a complete set of components, which can be grouped into three main categories: the structural framework, the hydroponic system, and the system electronics integration. Table 2 provides a detailed breakdown of all materials and components necessary for assembly and operation, including their specifications, unit costs, and suppliers. The total cost analysis indicates that, while an initial investment is required, the system remains cost-effective and suitable for implementation in most laboratory environments. This affordability also facilitates meaningful benchmarking against conventional upstream platforms, such as microbial, yeast, or mammalian cell cultivation systems. Optional equipment and accessories that can be incorporated into the hydroponic greenhouse system are listed in Table S2. These items may be used to validate and monitor operational variables of the greenhouse.

**Table 2:** Bill of Materials for the hydroponic greenhouse. A more detailed breakdown of the bill of material can be found in Section 5 and in Table S1.

Component	Number	Unit Cost	Total Cost	Supplier	Material Type
Silver Aluminium Anodized Profile Strut, 30 × 30 mm, 6 mm Groove, 2 m length	4	£35.75	£143.00	RS Components	Metal
Silver Aluminium Anodized Profile Strut, 30 × 30 mm, 6 mm Groove, 1 m length	1	£18.72	£18.72	RS Components	Metal
Cube Connector, 3-way, 30 mm Profile, 6 mm Groove	8	£11.70	£93.60	RS Components	Metal
Cube Connector, 2-way, 30 mm Profile, 6 mm Groove	4	£8.20	£32.80	RS Components	Metal
M6 T-slot Nut, Connecting Component, Strut profile 30 mm, 8 mm Groove	5	£7.08	£35.40	RS Components	Metal
Black Plastic Handle, 50 mm × 25 mm × 137 mm (pack of 2)	1	£10.67	£10.67	RS Components	Polymer
Standoff, M3 × M3 Thread, 35 mm Body, POM,Female/Female (pack of 10)	3	£7.38	£22.14	RS Components	Polymer
Nitrile Rubber O-Ring, 21 mm × 25 mm (pack of 50)	1	£4.81	£4.81	RS Components	Polymer
Acrylic Thick Sheet, 6 mm, 1000 × 1000 mm	1	£89.65	£89.65	Direct Plastics	Polymer
Acrylic Thick Sheet, 5 mm, 1000 × 500 mm	1	£39.22	£39.22	Direct Plastics	Polymer
Acrylic Thick Sheet, 4 mm, 1000 × 500 mm	1	£31.38	£31.38	Direct Plastics	Polymer
Aluminium Bar, 12 mm Diameter, 1000 mm long	1	£9.77	£9.77	Metals4U	Metal
Aluminium Plate, 8 mm Thick, 200 × 100 mm	1	£16.63	£16.63	Aluminium Warehouse	Metal
Resin 3D-Printed Components	1	£10.00	£10.00	N/A	Polymer
Filament 3D-Printed Components	1	£15.00	£15.00	N/A	Polymer
Assorted Screw and Nuts, Stainless	1	£33.23	£31.23	Westfield Fasteners	Metal
Hydraulic 1/2″ Ball Valve Male/Female	1	£10.67	£10.67	RS Components	Metal
2000 W LED Plant Grow Light	1	£84.00	£84.00	Koscheal	Electrical
Submersible Water Pump (450 L h^−1^)	1	£12.99	£12.99	Decdeal	Electrical
Blackout Window Film, 900 × 2000 mm	1	£19.99	£19.99	VSDUO	Polymer
AB1 Liquid Adhesive, 250 ml	1	£18.91	£18.91	RS Components	Adhesive
Dow Corning 786 Transparent Silicone Sealant 310 ml	1	£23.70	£23.70	RS Components	Sealant
Plug‑in digital timer	1	£7.00	£7.00	Ryman	Electrical
		**TOTAL**	**£781.28**		

## 5 Build instructions

This section provides step-by-step instructions for constructing the small-scale hydroponic greenhouse, from the mechanical frame and tray assembly to the installation of the lighting components. All parts referenced below correspond to the elements listed in the Bill of Materials summary (Table 2), and safety considerations for each section are provided in the Supplementary Information (Table S3).

### 5.1 Frame assembly

The frame assembly (Fig. 2) serves as the primary structural support for the lighting system, accommodating both fixed and moving components, including the lamp lifting mechanism. Constructing this frame requires aluminium beams (SA01-P01 to SA01-P05), along with joining plates, brackets, pulleys, and connectors (Table 3).

**Table 3:** Full detail of the material necessary to assembly the frame.

Item No.	Part No.	Description	Qty	Manufacturing Method/Notes	
1	SA01-P01	Frame Beam L = 760 mm	4	Band Saw cut to length, M8 Tap both ends	
2	SA01-P02	Frame Beam L = 450 mm	4	Band Saw cut to length, M8 Tap both ends	
3	SA01-P03	Frame Beam L = 440 mm	4	Band Saw cut to length, M8 Tap both ends	
4	SA01-P04	Frame Beam L = 360 mm	4	Band Saw cut to length, M8 Tap both ends	
5	SA01-P05	Frame Beam L = 225 mm	2	Band Saw cut to length, M8 Tap both ends	
6	SA01-P06	Beam Joining Plate	8	Laser Cut	
7	SA01-P07	Rachet Block Bracket	1	Filament 3D Print – Bambu	
8	SA01-P08	Guide Bar Position Bracket Long	1	Filament 3D Print – Bambu	
9	SA01-P09	Guide Bar Position Bracket Short	1	Filament 3D Print – Bambu	
10	SA01-P10	Lifting Line Tie Off Block	1	Filament 3D Print – Bambu	
11	SA01-P11	Lifting Line Guide Bar	2	Lathe Turning	
12	SA01-P12	Lamp Mounting Cable Tension Plate	1	CNC Milling	
13	SA01-P13	Lifting Line Bracket for Lamp	1	Resin 3D Print – Formlabs	
14	SA01-P14	Lift Line Pulley	3	Lathe Turning	
15	B01.3	Rachet Block	1	As supplied	
16	B01.4	Lifting Line	1	As supplied	
17	B03	Corner Connector 3 Way	8	As supplied	
18	B04	Corner Connector 2 Way	4	As supplied	
19	B05	M6 T-Nut	50	As supplied	
20	CSK08-020-SS	Countersunk Screw – M8 × 20 mm – A2 Stainless	32	As supplied	
21	BTN06-012-SS	Button Head Screw – M6 × 12 mm −A2 Stainless	40	As supplied	
22	BTN06-016-SS	Button Head Screw – M6 × 16 mm −A2 Stainless	10	As supplied	
23	BTN05-016-SS	Button Head Screw – M5 × 16 mm −A2 Stainless	4	As supplied	
24	BTN05-040-SS	Button Head Screw – M5 × 40 mm −A2 Stainless	2	As supplied	
25	BTN05-045-SS	Button Head Screw – M5 × 45 mm – A2 Stainless	1	As supplied	
26	CAP02.5–020-SS	Socket Cap Screw – M2.5 × 20 mm – A2 Stainless	4	As supplied	
27	NYL05-SS	Nyloc Nut – M5 – A2 Stainless	3	As supplied	

Prior to assembly, all aluminium frame beams (Items 1–5 in the Bill of Materials) must be tapped at both ends with an M8 × 25 mm thread to allow secure fastening. The assembly begins by forming the rectangular base using the longer beams (SA01-P01 and SA01-P03), with corners secured by 3-way and 2-way connectors (Items 17 and 18). Tighten all M8 bolts on these connectors to 18–22 N m to ensure structural stability. The upper rectangular frame is then constructed using the same method, mirroring the base, and connected to it with vertical beams (SA01-P02 and SA01-P04) to create the main cubic structure. Beam joining plates (SA01-P06) are added to the upper section to reinforce the frame and provide mounting points for the lifting mechanism, with M8 bolts also tightened to 18–22 N m. The 3D-printed ratchet block bracket (SA01-P07) is mounted on the upper right beam using M6 hardware tightened to 8–10 N m, and the ratchet block (Item 15) is fixed onto it with four M2.5 × 20 mm socket cap screws (Item 26) tightened to 0.4–0.6 N m, replacing the original screws. Guide bar position brackets (SA01-P08 and SA01-P09) are then attached to the top frame, followed by the installation of two lifting line guide bars (SA01-P11) to maintain cable alignment during operation. The lifting line (Item 16) is threaded through the system by tying one end to the tie-off block (SA01-P10), routing it across the guide bars and pulleys (SA01-P14), and terminating at the ratchet block. Finally, the lamp mounting tension plate (SA01-P12) and lifting line bracket (SA01-P13) are assembled to form the moving carriage that supports the LED lamp, which moves vertically when the lifting line is tensioned or released.

### 5.2 Tank assembly

The tank assembly forms the central hydroponic unit where the plant modules and nutrient solution are contained (Fig. 3). It is assembled from five custom-cut panels (Table 4, Items 1–5) and includes a drainage manifold. The assembly relies on sealing to ensure watertight operation under repeated filling and draining cycles.

**Table 4:** Full detail of the materials necessary to assembly the tank.

Item No.	Part No.	Description	Qty	Made From Shopping List Item No.	Manufacturing Method/Notes
1	SA02-P01	Tray Long Side Panel	2	12	Laser Cut
2	SA02-P02	Tray Deep End Panel	1	12	Laser Cut
3	SA02-P03	Tray Shallow End Panel	1	12	Laser Cut
4	SA02-P04	Tray Base Panel with Drain	1	12	Laser Cut
5	SA02-P05	Tray Slope Panel	1	12	Laser Cut
6	SA02-P06	Manifold for Ball Valve	1	19	Resin 3D Print – Formlabs
7	B06	O-Ring – 22 mm ID 2 mm CS	1	8	As supplied
8	B07	1/2″ Ball Valve Male/Female	1	9	As supplied

Begin by placing the SA02-P04 Tray Base Panel with Drain on a flat, level surface. Position the two SA02-P01 Tray Long Side Panels along the long edges of the base and apply a continuous layer of AB1 structural adhesive (RS part no. 144–406) to the contact surfaces. Clamp the panels securely. Next, bond the SA02-P02 Tray Deep End Panel and SA02-P03 Tray Shallow End Panel to the short edges of the base using the same method, ensuring correct orientation and perpendicular alignment. After all panels are clamped, apply an additional layer of AB1 adhesive along every internal joint to reinforce the structure. Allow the assembly to cure overnight.

Once the adhesive has cured, seal all internal seams with a continuous bead of silicone sealant (RS part no. 459-0232), smoothing to eliminate air pockets and ensure watertightness. Leave the tray for at least 48 h to allow complete curing before water testing. Complete the drainage system by inserting the SA02-P06 Manifold for Ball Valve through the drain opening in the base panel, fitting the B06 O-ring (22 mm ID, 2 mm CS) to seal the interface, and attaching the B07 ½″ male/female ball valve externally. This configuration provides a robust outlet for connection to a recirculation loop or rapid draining and cleaning.

### 5.3 Tray insert assembly

**Fig. 4:**
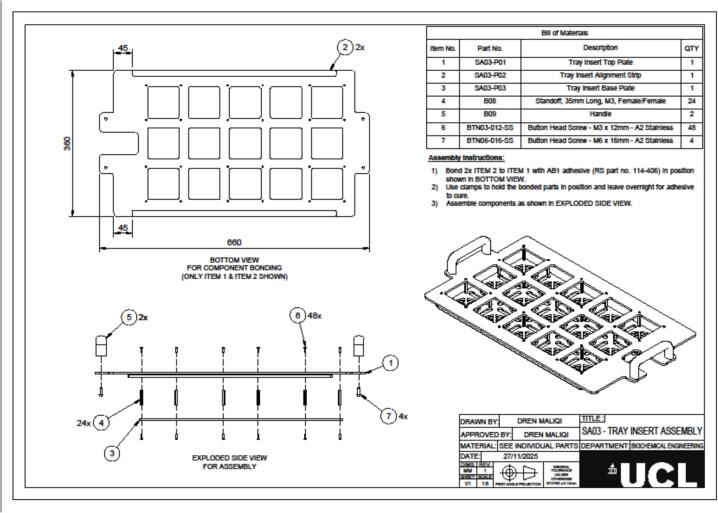
Technical drawing of the tray insert assembly showing bottom view for alignment strip bonding, exploded side view for standoff and handle installation, and complete assembled configuration with labelled components and bill of materials.

The tray insert is made of a top plate, an alignment strip, a base plate, and a set of standoffs and screws that hold everything together and create the support surface for the plant pots (Fig. 4, Table 5).

**Table 5:** Full detail of the materials necessary to assembly the tray.

Item No.	Part No.	Description	Qty	Made From Shopping List Item No.	Manufacturing Method/Notes
1	SA03-P01	Tray Insert Top Plate	1	13	Laser cut
2	SA03-P02	Tray Insert Alignment Strip	1	12	Laser cut
3	SA03-P03	Tray Insert Base Plate	1	14	Laser cut
4	B08	Standoff, 35 mm Long, M3, Female/Female	24	7	As supplied
5	B09	Handle	2	6	As supplied
6	BTN03-012-SS	Button Head Screw – M3 × 12 mm – A2 Stainless	48	27	As supplied
7	BTN06-016-SS	Button Head Screw – M6 × 16 mm – A2 Stainless	4	23	As supplied

Assembly begins by bonding the two SA03-P02 alignment strips to the underside of the SA03-P01 top plate using AB1 structural adhesive (RS part no. 114-406). Position the strips according to the bottom view shown in Fig. 4 so they align along the inner edges. After applying adhesive, clamp the strips securely and allow them to cure overnight. Once cured, assemble the tray insert by stacking the SA03-P03 base plate beneath the bonded top plate, ensuring all holes are aligned. Insert 24 standoffs (B08, M3 × 35 mm) between the two layers and secure them with 48 stainless-steel button-head screws (M3 × 12 mm, part no. BTN03-012-SS), tightening to maintain rigidity and levelness. Finally, attach the two handles (B09) to the top plate using four M6 × 16 mm screws (part no. BTN06-016-SS), completing the assembly and providing a secure grip for lifting and placing the insert into the hydroponic tank. The assembled insert offers a stable, removable support for plant pots, facilitating easy cleaning and transplanting.

### 5.4 Fully assembled hydroponic system

**Fig. 5:**
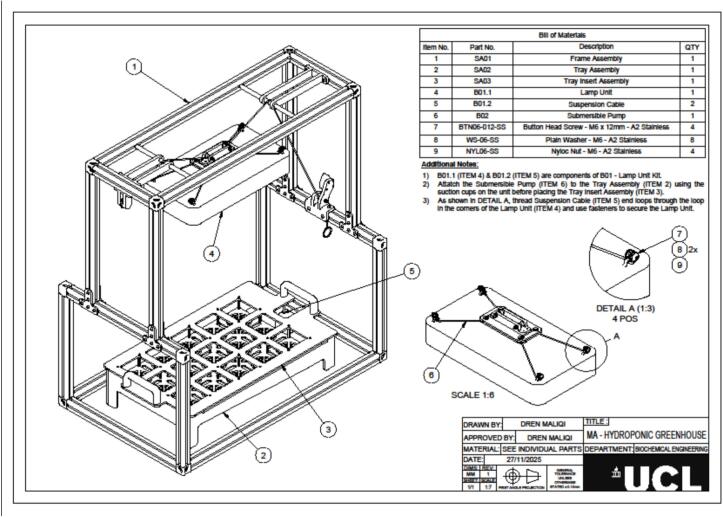
Isometric view of the hydroponic greenhouse showing the frame, tray assembly, lamp unit with suspension cables, and pump compartment. Detail A illustrates the lamp attachment points. The diagram serves as a visual guide for component positioning and assembly.

The exploded view of the hydroponic greenhouse provides a comprehensive breakdown of all structural and functional components, serving as a visual guide for assembly (Fig. 5). The illustration shows the frame assembly (SA01) forming the main structural support, with the tray assembly (SA02) positioned at the base to hold the plant insert. Above the tray, the lamp unit (SA04) is suspended using adjustable cables (B11), ensuring optimal light distribution for plant growth. The submersible pump (B12) is located within the pump compartment, enabling nutrient solution circulation through the hydroponic system. Detail A highlights the attachment points for securing the lamp unit to the frame using M6 button-head screws (BTN06-016-SS) and nyloc nuts (NYLO6-SS). This exploded view clarifies the relative positioning of each component, including the tray insert, lighting system, and irrigation hardware, ensuring accurate assembly and alignment for efficient operation.

## 6 Operation instructions

Once the cultivation system is fully assembled, it should be placed inside a conventional fume hood to maintain a protected microenvironment and ensure continuous air circulation. A dark, non-reflective film (Table 2, blackout window film) should be used to shield the hood, preventing external light from entering while also avoiding internal reflections from the LED lamp. This measure protects the plants from unwanted light exposure and reduces operator discomfort caused by the intensity of the LED illumination.

Before starting operations, verify that the drain siphon is closed (Fig. 3). Fill the hydroponic tray with the pre-mixed nutrient solution, including oxygen solution, prepared according to the optimised formulation used for *N. benthamiana*. Rockwool cubes containing the germinated seedlings are then inserted into the designated positions on the supporting grid. Once the plants are in place, activate the submersible pump to initiate recirculation of the nutrient solution within the tray.

The LED lamp is connected to a plug‑in digital timer, which is then connected to the power socket. The lamp height is adjusted to maintain a consistent distance of 25 cm from the plants canopy (see Fig. 5). The timer is programmed to deliver the desired photoperiod for optimal plant growth. *N. benthamiana* grows best under a light:night photoperiod of 16:8 h. The timer settings can be modified to adjust the photoperiod as required. The nutrient solution must be changed once a week, with fresh oxygenated solution added daily.

At the end of 4 weeks of growth, the rockwool cubes are removed to proceed with the collection of the leaves (which will then be processed for the extraction and purification of the product of interest). Emptying the liquid contained in the tank occurs by opening the underlying siphon, which pours the contents into a sink. Once the tray has been emptied, it can be cleaned along the grid with detergents and bleach. The system is then ready for the next cultivation cycle.

## 7 Validation and characterization

To validate the performance of the hydroponic greenhouse, four independent cultivation batches of *N. benthamiana* plants were grown and monitored over a complete four-week cycle. Six quantitative parameters were measured: (i) days to cotyledon emergence, (ii) germination efficiency, (iii) plant height, (iv) total leaf area, (v) light intensity distribution, and (vi) temperature and relative humidity stability. A set of qualitative observations, including leaf coloration, root health and flowering time, was also recorded to assess overall plant health status.

### 7.1 Light distribution

**Fig. 6:**
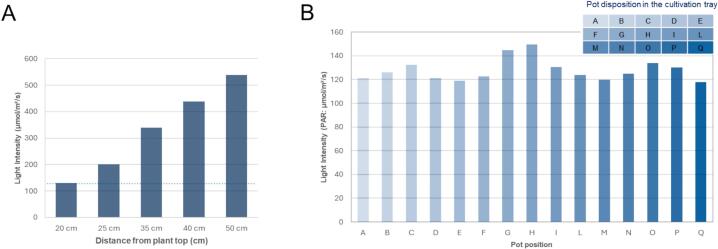
Light intensity characterisation of the LED lamp in the small-scale hydroponic greenhouse, measured using a lux metre. A lamp–canopy distance of 25 cm provided optimal PAR values (100–150 µmol m⁻^2^ s⁻^1^) for *N. benthamiana* growth (A), with uniform illumination across the cultivation area and a stable spectrum within the 400–700 nm range (B).

Light affects plant growth and transient gene expression, where plants grown under controlled lighting, light intensity and distribution may impact protein yields. Therefore, a LuxMeter was used to measure light intensity at the surface of each rockwool cube (Fig. 6). Prior to the validation experiments, the LED lamp was characterised at multiple canopy distances to identify the optimal photosynthetic photon flux density (PPFD) for *N. benthamiana* vegetative growth (Fig. 6A). These measurements showed that a lamp–canopy distance of 25 cm consistently produced PAR values within the optimal range of 100–150 µmol m⁻^2^ s⁻^1^, and this configuration was therefore adopted for all batches. At the fixed operational distance of 25 cm, the LED unit delivered a spectrum within the PAR range 400–700 nm and provided stable light intensity across the cultivation area (Fig. 6B). The measurements confirmed uniform illumination across all plant positions, ensuring that each plant received comparable illumination (average 127.9 ± 9.3 µmol m⁻^2^ s⁻^1^, max. 149.7 µmol m⁻^2^ s⁻^1^, and min. 117.9 µmol m⁻^2^ s⁻^1^). This validated both the choice of the LED system and the structural layout of the frame, which maintains the recommended light–canopy distance.

### 7.2 Temperature and humidity stability

**Fig. 7:**
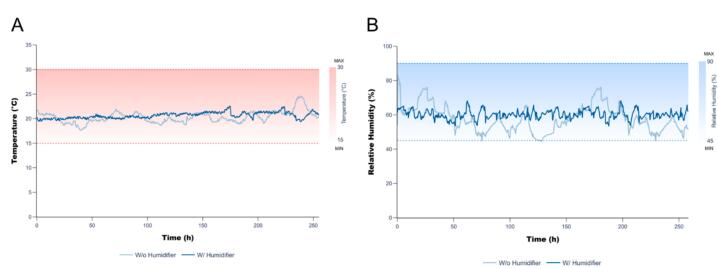
Temperature (A) and relative humidity (B) profiles over time, with and without the humidifier, in the small-scale hydroponic greenhouse. Temperature and humidity were recorded with an ElitechLog V8.0 data logger. Shaded regions indicate the acceptable ranges for optimal *N. benthamiana* growth.

Probes placed inside the nutrient-solution tank and on the external frame continuously monitored temperature and relative humidity throughout the experiments (Fig. 7). *N. benthamiana* grows optimally between 23 and 25 °C and approximately 60% relative humidity. Prior to environmental optimisation, temperature fluctuated between 19 and 26 °C (Fig. 7A), and relative humidity ranged from 45 to 81% (Fig. 7B), likely influenced by airflow from the fume hood. Introducing a humidifier reduced these fluctuations, resulting in more stable conditions (20.5 ± 1.5 °C and 57.3 ± 7.6% RH) confirming the benefit of this optimisation step.

Overall, the system performed reliably, providing consistent, reproducible, and physiologically suitable growth conditions for *N. benthamiana*. All key parameters were measurable, monitored, and manually adjustable within the hardware. The greenhouse setup therefore offers a scalable and resource-efficient platform that supports alignment with good cultivation practice principles while remaining accessible for early-stage PMF research.

### 7.3 Germination validation

Seeds were germinated in rockwool plugs under controlled conditions inside the fume hood, at 27 °C and 90% relative humidity, to allow the outer protective layer of the seed to break down and the first cotyledonal leaf to emerge [19]. Two germination metrics were evaluated: the number of days from sowing to cotyledon emergence and the percentage of seeds germinating out of the total sown (Table 6). Across the four batches, both metrics showed consistent and reproducible behaviour with only minor variability, reflecting the expected biological variation of living plants. Within one week, root systems had developed sufficiently to allow transplantation into the larger rockwool cubes positioned in the greenhouse tray.

**Table 6:** Germination performance across four seed batches (n = 4), showing average days to cotyledon emergence and percentage of seeds successfully germinated under controlled conditions (27 °C, and 90% relative humidity).

Batch	Days to Germination	Seeds Germinated (%)
A	5	61.5
B	6	60.0
C	5	62.1
D	5	62.0

### 7.4 Plant growth characterization

**Fig. 8:**
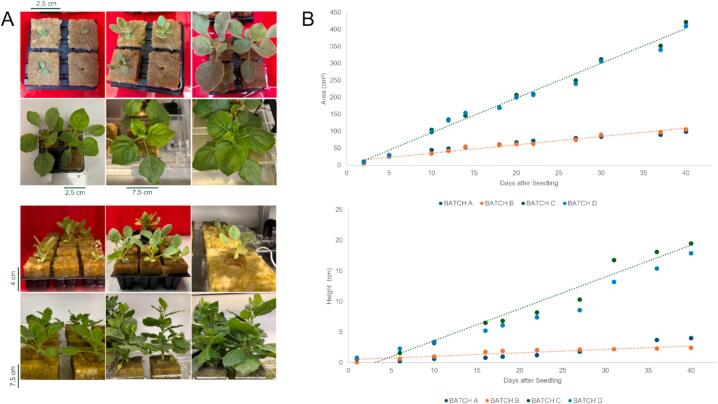
*N. benthamiana* growth in the developed hydroponic greenhouse. (A) Plant development from seedling to mature plant. Picture with red background are the initial stages of the growth, from germination to the point where the *N. benthamiana* sprouts are transferred to the rockwool cubes and into the greenhouse. (B) Quantitative comparison of total leaf area, in cm^2^ (top) and plant height in cm (bottom) as a function of days after seeding for four batches (A–D). Batches A and B, cultivated without a humidifier, show limited and inconsistent growth. Batches C and D, grown under monitored humidity, display markedly improved development, reaching ∼350–450 cm^2^ leaf area and ∼15–25 cm height by the end of the cycle. Trend lines highlight the growth patterns. (For interpretation of the references to colour in this figure legend, the reader is referred to the web version of this article.)

Plant height and total leaf area were recorded every 2–3 days throughout the growth cycle (Fig. 8A). Plant height was measured from the base of each plant, while leaf area was quantified from top-view images processed in ImageJ by thresholding the leaf outline and calculating the corresponding pixel area. Plants grown in the greenhouse without a humidifier reached a maximum height of 4 cm and a leaf area of 106 cm^2^ after 40 days (Fig. 8B; batches A and B). Although the continuous airflow minimised contamination risk, the absence of a humidifier introduced dry, cool air that reduced temperatures below the optimal growth range. Following the introduction of a humidifier, batches C and D showed markedly improved and more uniform growth (Fig. 8B; batches C and D). Plants reached a maximum of 23 cm in height and approximately 420 cm^2^ in total leaf area by the end of the growth cycle (total plant mass: 623 g, and leaf mass: 489 g), confirming that the optimised environmental conditions supported healthy and consistent development.

## 8 Conclusions

The small-scale hydroponic greenhouse developed in this study successfully demonstrated its ability to provide supervised, reproducible, and physiologically suitable conditions for *Nicotiana benthamiana* cultivation. Validation across four independent batches confirmed the system’s robustness. Following environmental optimisation with a humidifier, temperature and relative humidity remained within suitable ranges (23–25 °C and ∼60% relative humidity), eliminating fluctuations previously caused by the fume hood airflow. This improved stability supported consistent plant development, with final heights of 20–25 cm and leaf areas of approximately 400 cm^2^ (total leaf mass of 489 g), meeting the requirements for transient-expression workflows in Plant Molecular Farming (PMF).

Light distribution measurements using PAR sensors confirmed uniform illumination at a fixed lamp–canopy distance of 25 cm, delivering the desired flux density (100–150 µmol m⁻^2^ s⁻^1^). Germination remained reproducible across batches, achieving >60% efficiency with predictable cotyledon emergence timelines. These findings collectively demonstrate that the platform supports homogeneous and reliable plant development, a key prerequisite for consistent biopharmaceutical production.

In addition to supervised operation of the system, routine use revealed a small number of recurring practical issues, for which straightforward mitigation strategies were identified (Table 7). These operational obervations provide a concise troubleshooting guide to support reproducible performance across experimental campaigns.

**Table 7:** Troubleshooting guide for the hydroponic greenhouse system.

Issue	Observation	Possible cause	Corrective actions
Tray leakage	Visible seepage, wet surface under tray	Incomplete adhesive coverage, glue not cured, glue clumps preventing proper sealing	Empty and dry tray, then re-apply uniform adhesive, allow full curing, remove glue clumps to avoid residue and contamination hotspots
Corrosion of screws and nuts	Rust, weakened joints, difficulty tightening	Use of non-resistant screws and nuts, elevated humidity	Replace with specified corrosion-resistant hardware (e.g. stainless steel, per instructions)
Excessive evaporation	Rapid solution loss, rising conductivity, frequent top-ups needed	Low humidity, large plants with high transpiration, high airflow in the fume hood	Use humidifier to stabilise RH, increase monitoring frequency. Automatic top-up system can be considered
Algae growth in reservoir	Green coloration in the tray, slimy surfaces	Light reaching reservoir, insufficient aeration, low solution temperature	Aerate solution daily either by providing oxygenation solution or use an air pump, maintain solution at 25 °C using heater in solution tray, cover reservoir to block light. Optionally, use UV lamp if properly enclosed and risk-assessed
Temperature or humidity instability	Slow growth, non-uniform morphology, inconsistent batch performance	Poor sealing of fume hood enclosure, uncalibrated sensors, insufficient heating/humidification	Seal enclosure gaps, recalibrate sensors and maintain validated ranges (23–25 °C and ∼60% RH)

This work delivers an open-source, laboratory-scale hydroponic greenhouse that addresses a critical gap in PMF research infrastructure. With a build cost of £781 and a modular, accessible design, the system enables supervised and reproducible cultivation of *N. benthamiana* without reliance on specialised growth facilities. Its scalability and ease of integration into standard laboratory environments allow researchers to accelerate prototyping of plant‑based biomanufacturing workflows while reducing dependence on costly large‑scale systems. Ultimately, this hardware provides a sustainable and affordable platform that accelerates upstream process development and early-stage PMF experimentation.

The small-scale hydroponic greenhouse successfully addresses the need for an affordable, reproducible, and supervised cultivation system for PMF applications. Validation confirmed that the platform supports consistent environmental conditions for suitable *N. benthamiana*, enabling uniform growth and facilitating transient-expression workflows. Its modular design, low cost, and compatibility with standard laboratory infrastructure make it a practical tool for both academic and industrial research settings.

## Ethics statements

The work did not involve any human or animal subjects, nor data from social media platforms.

## CRediT authorship contribution statement

**Teresa Iucci:** Writing – original draft, Methodology, Investigation, Formal analysis, Conceptualization. **Dren Maliqi:** Writing – review & editing, Methodology, Conceptualization. **Sara Sousa Rosa:** Writing – review & editing. **Marco P.C. Marques:** Writing – review & editing, Supervision, Funding acquisition, Conceptualization.

## Declaration of competing interest

The authors declare that they have no known competing financial interests or personal relationships that could have appeared to influence the work reported in this paper.
