# Supplementary material for: A compact, modular and low‑cost hydroponic greenhouse
Source: HardwareX. 2026 Apr 26;26:e00777. doi: 10.1016/j.ohx.2026.e00777 (PMC13141774; doi:10.1016/j.ohx.2026.e00777)
Supplement: Supplementary Data 1 [file mmc1.pdf]

A compact, modular and low-cost hydroponic greenhouse

*Teresa Iucci[1,3], Dren Maliqi[2], Sara Sousa Rosa[1], Marco PC Marques[1]**

[1] Department of Biochemical Engineering, University College London, Gordon Street, London, WC1E 6BT, United Kingdom

[2] High Precision Design and Fabrication Facility, University College London, Torrington Place, London, WC1E 7JE, United Kingdom

[3] Department of Chemistry and Pharmaceutical Technologies, University La Sapienza, Piazzale Aldo Moro 5, 00185, Rome, Italy

Corresponding author's email address and Twitter handle
marco.marques@ucl.ac.uk, twitter (X): @marcopcmarques

Table of content:

Tabel S1. Bill of materials for the hydroponic greenhouse

Table S2. Bill of materials of optional equipment and accessories that can be incorporated into the hydroponic greenhouse system

Table S3. Health and safety risk considerations associated with the assembly of each component of the small-scale hydroponic greenhouse.

Tabel S1. Bill of materials for the hydroponic greenhouse

Item No.	*Description*	*Supplier*	*Supplier Part No.*	*QTY*	*Unit Cost*	*Sub Total*
1	RS PRO Silver Aluminium, Anodized Profile Strut, 30 x 30 mm, 8mm Groove, 2m Length	RS Components	264-7863	4	£35.75	£143.00
2	RS PRO Silver Aluminium, Anodized Profile Strut, 30 x 30 mm, 8mm Groove, 1m Length	RS Components	264-7862	1	£18.72	£18.72
3	RS PRO Cube Connector, 3 Way, Strut Profile 30 mm	RS Components	767-5575	8	£11.70	£93.60
4	RS PRO Cube Connector, 2 Way, Strut Profile 30 mm	RS Components	767-5572	4	£8.20	£32.80
5	RS PRO M6 T-Slot Nut, Connecting Component, Strut Profile 30 mm, Groove Size 8mm (Bag of 10)	RS Components	276-8170	5	£7.08	£35.40
6	RS PRO Black Plastic Handle 50 mm Height, 25mm Width, 137mm Length (Pack of 2)	RS Components	456-563	1	£10.67	£10.67
7	HIROSUGI Standoff, M3 x M3 Thread, 35mm Body, POM, Female/Female (Bag of 10)	RS Components	664-3240	3	£7.38	£22.14
8	RS PRO Nitrile Rubber O-Ring O-Ring, 21mm Bore, 25mm Outer Diameter (Bag of 50)	RS Components	196-4881	1	£4.81	£4.81
9	RS PRO Chrome Plated Brass Hydraulic Ball Valve G 1/2	RS Components	486-356	1	£10.67	£10.67
10	RS PRO Liquid Adhesive AB1, 250 ml	RS Components	144-406	1	£18.91	£18.91
11	Dow Corning 786 Transparent Sealant Paste 310 ml Cartridge	RS Components	459-0232	1	£23.70	£23.70
12	Acrylic 6mm Thick Sheet 1000mm x 1000mm	Direct Plastics	N/A	1	£89.65	£89.65
13	Acrylic 5mm Thick Sheet 1000mm x 500mm	Direct Plastics	N/A	1	£39.22	£39.22
14	Acrylic 4mm Thick Sheet 1000mm x 500mm	Direct Plastics	N/A	1	£31.38	£31.38
15	12mm Diameter Aluminium Bar 1000mm long	Metals 4u	102190	1	£9.77	£9.77
16	8mm Thick Aluminium Plate 200mm x 100mm	Metals 4u	115113	1	£16.63	£16.63
17	Decdeal 450L/H 6W Submersible Water Pump	Amazon UK	N/A	1	£12.99	£12.99

18	KOSCHEAL KS2000 LED Grow Light	Amazon UK	N/A	1	£84.00	£84.00
19	Approximate Cost of Resin 3D Printed Parts - Formlabs	N/A	N/A	1	£10.00	£10.00
20	Approximate Cost of Filament 3D Printed Parts - Bambu	N/A	N/A	1	£15.00	£15.00
21	Countersunk Screw - M8 x 20mm - A2 Stainless (Custom pack of 40)	Westfield Fasteners	WF2588	1	£8.78	£8.78
22	Button Head Screw - M6 x 12mm - A2 Stainless (Custom pack of 50)	Westfield Fasteners	WF2254	1	£5.24	£5.24
23	Button Head Screw - M6 x 16mm - A2 Stainless (Custom pack of 20)	Westfield Fasteners	WF2255	1	£3.79	£3.79
24	Button Head Screw - M5 x 16mm - A2 Stainless (Pack of 6)	Westfield Fasteners	WF2245	1	£1.66	£1.66
25	Button Head Screw - M5 x 40mm - A2 Stainless (Pack of 3)	Westfield Fasteners	WF2250	1	£1.36	£1.36
26	Button Head Screw - M5 x 45mm - A2 Stainless (Pack of 2)	Westfield Fasteners	WF2251	1	£1.05	£1.05
27	Button Head Screw - M3 x 12mm - A2 Stainless (Custom Pack of 50)	Westfield Fasteners	WF2228	1	£2.81	£2.81
28	Socket Cap Screw - M2.5 x 20mm - A2 Stainless (Pack of 6)	Westfield Fasteners	WF2341	1	£1.54	£1.54
29	Washer M6 - Stainless (Pack of 8)	Westfield Fasteners	WF21	1	£1.39	£1.39
30	Nyloc Nut M5 - Stainless (Pack of 4)	Westfield Fasteners	WF1297	1	£1.94	£1.94
31	Nyloc Nut M6 - Stainless (Pack of 6)	Westfield Fasteners	WF1291	1	£1.67	£1.67
32	Plug-in Digital Timer	Ryman	N/A	1	£7.00	£7.00
33	Blackout Window Film – 900x2000 mm	VSDUO	N/A	1	£19.99	£19.99
					Total	***£781.28***

Table S2. Bill of materials of optional equipment and accessories that can be incorporated into the hydroponic greenhouse system

Description	***Supplier***	***Part number***	***Total Cost***
Light meter	RS Components	RS-3809	£92.48
ElitechLog V8.0	Elitech	RC-5	£39.99
Cling film 450mm x 305m	Appleton Woods Ltd	F GC348	£16.58
Pegdev 4m 1000G black heavy polythene plastic sheeting garden	PDL Garden Services Ltd	POLYBLK	£12.00
pH meter portable	Mettler - Toledo	662-2007	£299.30
Conductivity meter	Mettler - Toledo	30266887	£489.23
Rockwool Cubes (276 cubes)	Cultiene	10001030	£121.49
Rockwool Propagation plugs (1286 plugs)	Cultiene	10000251	£152.99
Hydro Grow nutrient solution (1L)	Growth Tecnhology	106281	£10.99
Liquid Oxygen (1L)	Growth Tecnhology	104005	£8.95
Liquid Silicon (1L)	Growth Tecnhology	104002	£11.95
5 L Top Fill Humidifier	Bear Eletric Appliance Ltd	JSQ-F50D2	£29.99
		Total	*£1,285.94*

Table S3. Health and safety risk considerations associated with the assembly of each component of the small-scale hydroponic greenhouse. This table summarises the main hazards, recommended protective measures, and operational precautions for each construction stage.

**Section – Assembly stage**	**Health and Safety Considerations**
5.1 Frame Assembly	During assembly, ensure that all tapped threads are free of debris and burrs to prevent compromised fastening and structural integrity. Operators must wear appropriate personal protective equipment (PPE), including safety glasses and cut-resistant gloves, when performing cutting, tapping, or handling aluminium profiles. All connectors and fasteners should be tightened to the specified torque values using a calibrated torque wrench to avoid loosening or over-tightening, which may lead to structural failure. When routing the lifting line, maintain hands and fingers clear of pulleys, ratchet blocks, and tensioning components to mitigate the risk of pinch injuries. Prior to operating the lifting mechanism, verify that the ratchet block is securely mounted and that the cable is correctly aligned to prevent sudden load release or uncontrolled movement. After installation, conduct routine inspections for loose connections, wear on moving parts, and signs of fatigue in structural components. Any damaged or worn parts should be replaced immediately to maintain operational safety and reliability.
5.2 Tank Assembly	During assembly, wear appropriate PPE to prevent contact with adhesives and sealants. Work in a well-ventilated area or under a fume hood to minimize inhalation of volatile compounds. Ensure clamps are securely positioned to avoid sudden release and keep hands clear of pinch points during tightening. Allow adhesives and sealants to fully cure before water testing to prevent structural failure or leaks. Use proper tools for valve installation without applying excessive force to avoid damage or injury. Finally, conduct leak tests only after complete curing and avoid pressurized systems until structural integrity is confirmed.

5.3 Tray insert Assembly	The adhesive bonding should be performed in a well-ventilated area or under a fume hood to avoid inhalation of fumes. Wear chemical-resistant gloves and safety goggles to prevent skin and eye contact with adhesives and sealants.
5.4. Fully assembled hydroponic system	When relocating components of the small-scale hydroponic greenhouse, only the plant support grid should be moved during normal operation. All other components must be handled only once the tray has been fully drained of nutrient solution. Components should be moved individually, i.e. first the plant support grid, followed by the empty tray, and finally the frame. Prior to any movement, all sensors and the pump must be removed, and all electrical cables, including those connected to the LED lamp, must be fully disconnected to avoid entanglement or electrical hazards. Appropriate manual-handling techniques must be used at all times, taking account of the component weights: frame (10.2 kg), LED system (2.5 kg), tray (2.7 kg empty). and plant support grid (2 kg). The working area should be kept clear and dry to minimise slip and trip risks.
